# Comprehensive Analysis of Cuproptosis-Related Genes in Immune Infiltration and Prognosis in Melanoma

**DOI:** 10.3389/fphar.2022.930041

**Published:** 2022-06-28

**Authors:** Haozhen Lv, Xiao Liu, Xuanhao Zeng, Yating Liu, Canjing Zhang, Qi Zhang, Jinhua Xu

**Affiliations:** ^1^ Department of Dermatology, Huashan Hospital, Fudan University, Shanghai, China; ^2^ Institutes of Biomedical Sciences, Fudan University, Shanghai, China; ^3^ Shanghai Institute of Dermatology, Shanghai, China

**Keywords:** ion-driven cell death, melanoma, LIPT1, prognosis, immune infiltration

## Abstract

Skin cutaneous melanoma (SKCM, hereafter referred to as melanoma) is the most lethal skin cancer with increasing incidence. Regulated cell death plays an important role in tumorigenesis and serves as an important target for almost all treatment strategies. Cuproptosis is the most recently identified copper-dependent regulated cell death form that relies on mitochondria respiration. However, its role in tumorigenesis remains unknown. The correlation of cuproptosis-related genes with tumor prognosis is far to be understood, either. In the present study, we explored the correlation between cuproptosis-related genes with the prognosis of melanoma by accessing and analyzing a public database and found 11 out 12 genes were upregulated in melanoma tissues and three genes (LIPT1, PDHA1, and SLC31A1) have predictive value for the prognosis. The subgroup of melanoma patients with higher cuproptosis-related gene expression showed longer overall survival than those with lower gene expression. We chose LIPT1 for further exploration. LIPT1 expression was increased in melanoma biopsies and was an independent favorable prognostic indicator for melanoma patients. Moreover, LIPT1 expression was positively correlated with PD-L1 expression and negatively associated with Treg cell infiltration. The melanoma patients with higher LIPT1 expression showed longer overall survival than those with lower LIPT1 expression after receiving immunotherapy, indicating the prognostic predictive value of LIPT1. Finally, a pan-cancer analysis indicated that LIPT1 was differentially expressed in diverse cancers as compared to normal tissues and correlated with the expression of multiple immune checkpoints, especially PD-L1. It could serve as a favorable prognosis indicator in some cancer types. In conclusion, our study demonstrated the prognostic value of cuproptosis-related genes, especially LIPT1, in melanoma, and revealed the correlation between LIPT1 expression and immune infiltration in melanoma, thus providing new clues on the prognostic assessment of melanoma patients and providing a new target for the immunotherapy of melanoma.

## Introduction

Skin cutaneous melanoma (SKCM, hereafter referred to as melanoma), arising from the malignant transformation of melanocytes, is the most aggressive and deadliest type of skin cancer (Huang and Zappasodi, 2022). Although it accounts for about 1% of all skin cancers, it is responsible for about 80% of the deaths from these skin cancers (Banach et al., 2021; Moyers and Glitza Oliva, 2021). Worse still, its annual incidence kept on increasing over the past several decades (Muzumdar et al., 2021; Welch et al., 2021). The interaction between genetic susceptibility and environmental exposures contributes to the onset and development of melanoma (Dzwierzynski, 2021). Ultraviolet exposure and high tumor mutation burden are supposed to be the most common risk factors in melanoma (Huang and Zappasodi, 2022).

The localized or regional melanoma can be removed by surgery (Morton et al., 2014; Curti and Faries, 2021). However, patients with advanced melanoma can only be treated with systemic therapy (Curti and Faries, 2021). Dacarbazine was the standard chemotherapy for melanoma (Curti and Faries, 2021). However, the overall survival of the patients treated with dacarbazine was about 9.1 months, and the response rates were about 5% (Chapman et al., 2011; Robert et al., 2011). Melanoma harbored the highest genetic mutation burden among all solid tumors. Oncogenic mutations in BRAF were observed in about 40–50% of melanoma patients (Sullivan and Flaherty, 2013). Vemurafenib, a BRAF inhibitor, became the first approved targeted drug for the treatment of melanoma bearing oncogenic BRAF gene mutation (Curti and Faries, 2021). Although it can improve the median overall survival to 15.9 months, the median duration of response was 6.7 months, indicating the primary or acquired resistance to these targeted therapies (Sosman et al., 2012). Though the combination of BRAF inhibitor and MEK inhibitor can improve the response rates, the median progression-free survival, and overall survival, the acquisition of resistance remains inescapable (Larkin et al., 2014; Long et al., 2014; Robert et al., 2015a; Curti and Faries, 2021). Therefore, it is necessary to fully understand the landmarks of melanoma to reveal new targets.

Immune checkpoints, such as cytotoxic T-lymphocyte antigen-4 (CTLA-4) and programmed cell death-1 (PD-1), are surface proteins primarily expressed on T cells. They inhibit the initiation, duration, and magnitude of the immune responses when interacting with their ligands expressed on the antigen-presenting cells (Su and Fisher, 2016; Carlino et al., 2021). Immune checkpoint inhibitors (ICIs) restore the antitumor activity of cytotoxic T cells by blocking these immune checkpoints or their ligands (Barrios et al., 2020). Ipilimumab, an antibody against CTLA-4, was approved for the treatment of melanoma in 2011. It became the first ICI that received approval for tumor immunotherapy (Bagchi et al., 2021). It could improve the median overall survival to 19.9 months (Wolchok et al., 2017). The objective response rates to anti-CTLA-4 antibodies were about 10%–20% (Postow et al., 2015). With the quick development of ICIs, antibodies against PD-1 and PD-L1 were next approved for the treatment of melanoma. These inhibitors can improve the objective response to 30–40% with fewer adverse events (Robert et al., 2015b; Robert et al., 2015c; Postow et al., 2015). Even though the combination of anti-PD-1 and anti-CTLA-4 further improved the objective response rate to about 60%, it was accompanied by more adverse events (Postow et al., 2015; Wolchok et al., 2017). Therefore, finding predictive biomarkers for the response to ICI-based immunotherapy is ultimate to maximize the therapeutic benefits. PD-L1, IDO, IFNγ signatures, tumor-infiltrating immune cells, neoantigen burden, along with some other factors were regarded to be important biomarkers (Herbst et al ., 2014; Gibney et al., 2016; Darvin et al., 2018).

Regulated cell death (RCD), also known as programmed cell death (PCD), is generally regulated by signaling molecules and has unique biochemical, morphological, and immunological characteristics (Del Re et al., 2019; Tang et al., 2019). A growing number of RCD forms (apoptosis, necroptosis, autophagy, ferroptosis, pyroptosis, alkaliptosis, oxeiptosis, parthanatos, entotic cell death, netotic cell death, and lysosome-dependent cell death) has been identified to be involved in diverse pathological and physiological processes, such as homeostasis and tumorigenesis (Tang et al., 2019). The dysregulation of single or mixed forms of RCD was demonstrated to be involved in various kinds of diseases, including cancers (Linkermann et al., 2014; Gong et al., 2019). As the earliest founded and best-studied form of RCD, apoptosis is regarded as the target of almost all treatment strategies, including chemotherapies, radiotherapies, target therapies, and immunotherapies (Tang et al., 2019; Qi et al., 2022). However, resistance to apoptosis was, to a large extent, a major cause of the failure of these treatment strategies (Gong et al., 2019). Different forms of RCDs can be therapeutic alternatives to each other (Gong et al., 2019; Qi et al., 2022). Therefore, finding new forms of RCD is of utmost importance.

Copper is a kind of trace metals that was essential for life. Keeping the right amount of copper plays an important role in maintaining the function of all organisms and homeostasis. Copper lacking will damage the function of copper-binding enzymes, while copper accumulation will induce death in cells (Kahlson and Dixon, 2022). Recently, Peter et al. confirmed that copper itself, not copper ionophore was toxic to cells (Tsvetkov et al., 2022). The cuproptosis is a totally newly found cell death form that is different from other known death forms, including apoptosis, ferroptosis, and necroptosis (Tsvetkov et al., 2022). It is dependent on mitochondrial respiration instead of adenosine triphosphate (ATP) production (Tsvetkov et al., 2022). Moreover, they found seven genes (FDX1, LIAS, LIPT1, DLD, DLAT, PDHA1, and PDHB) conferred resistance to cuproptosis, while three genes (MTF1, GLS, and CDKN2A) sensitized the cells to cuproptosis through whole-genome CRISPR-Cas9 selection screen (Tsvetkov et al., 2022). Copper importers (SCL31A1) and copper exporters (ATP7A and ATP7B) are important factors in keeping the intracellular copper concentration. CTR1, coded by SCL31A1, was essential in the uptake of high-affinity copper (Lutsenko, 2010). It helps retrieve cooper from specific carriers (Moriya et al., 2008). Mutations in ATP7A and ATP7B genes were found to cause Menke’s disease and Wilson’s disease, respectively, which were characterized by block and accumulation of copper (Nevitt et al., 2012). Peter et al. indicated that overexpression of SCL31A1 and deletion of ATP7B could sensitize cuproptosis (Tsvetkov et al., 2022). However, the role of cuproptosis in the onset, development, and prognosis of tumors remains unknown. Moreover, since cuproptosis is a newly discovered form of RCD, its potential in being the target of immunotherapy is far to understand.

In our present study, we explored the correlation between cuproptosis-related genes with the prognosis of melanoma through accessing and analyzing a public database, wishing to provide new thoughts for the treatment and prognosis evaluation of melanoma. An overview of the research design was presented in Supplementary Figure S1.

## Materials and Methods

### Data Acquisition

RNA-sequencing expression profiles of 470 patients with SKCM were obtained from the TCGA dataset (https://portal.gdc.cancer.gov/projects/TCGA-SKCM). Samples of normal tissue were obtained from the GTEx data portal (https://www.gtexportal.org/home/datasets). GSE100050 and GSE114445 from the Gene Expression Omnibus (https://www.ncbi.nlm.nih.gov/geo/) database were downloaded and used to further validate LIPT1s expression level.

Cuproptosis-related genes were identified from the previous literature by Tsvetkov et al. (2022).

The protein expression level of LIPT1 in tumors compared to normal tissue was obtained from the Human Protein Atlas database (https://www.proteinatlas.org/).

### Subgroup Analysis

The “ConsensusClusterPlus” R package (v1.54.0) was used for the consistency analysis. The maximum number of clusters is six, and 80% of the total sample is drawn 100 times, clusterAlg = “hc”, innerLinkage = “ward.D2”. The gene expression heatmap retains genes with SD > 0.1. If the number of input genes is more than 1,000, it will extract the top 25% of genes after sorting the SD. Samples from the TCGA database were divided into two clusters on the basis of the cuproptosis-related genes level in SKCM patients.

Using the limma package in the R software to study the differentially expressed mRNA. “Adjusted *p* < 0.05 and |log2FC| > 2” were defined as the threshold for the differential expression of mRNAs between two clusters.

### Immune Score Analysis

To assess the reliable results of immune score evaluation, we used the “CIBERSORT . It is the latest algorithm of the “immunedeconv” package. All the analysis methods and R package used in immune score analysis were implemented by R (version 4.0.3) and software packages “ggplot2” and “pheatmap”.

### Survival Analysis

For Kaplan–Meier curves, *p* values and hazard ratio (HR) with 95% confidence interval (CI) were generated by log-rank tests and univariate cox proportional hazards regression. HR represents the hazard ratio of the low-expression sample relatives to the high-expression sample. HR > 1 indicates the gene is a risk factor, and HR < 1 indicates the gene is a protective factor. R packages “ggsrisk”, “survival”, “survminer”, and “timeROC” were implemented by R (version 4.0.3). *p* value < 0.05 was considered statistically significant.

Univariate and multivariate cox regression analyses were performed to identify the proper terms to build the nomogram. The forest was used to show the *p* value, HR, and 95% CI of each variable through the “forestplot” R package.

### Functional Enrichment Analysis

The data were analyzed by functional enrichment to clarify further the function underlying potential targets. To better understand the carcinogenesis of mRNA, “ClusterProfiler” package (version 3.18.0) in R was employed to analyze the GO function of potential targets and enrich the KEGG pathway. The R software ggplot2 package was used to draw boxplot; the R software “heatmap” package was used to draw the heatmap.

GSVA was run using “GSVA” (version 1.30.0) to ascertain the different pathways for analyzing the relation between LIPT1 and biological function in SKCM. An adjusted *p* < 0.05 was considered to indicate statistical significance between different subgroups by the “limma” package.

### Statistical Analysis

The statistical difference between the two groups was compared through the Wilcox test. Spearman correlation analysis was performed to evaluate the correlation between expression levels of LIPT1 with checkpoint-related genes. Kaplan-Meier survival analysis of the gene signature from TCGA dataset, comparison among different groups was made by log-rank test. The predictive efficiency of genes for overall survival (OS) was estimated using the receiver operating characteristic (ROC) curves by the “timeROC” package. Univariate and multivariate cox regression analyses were performed to identify the proper terms to build the nomogram Statistical analyses were performed using R software. *p*-value <0.05 was considered statistically significant.

## Results

### Cuproptosis-Related Genes That Were Differentially Expressed Between Melanoma and Normal Biopsies

**FIGURE 1:**
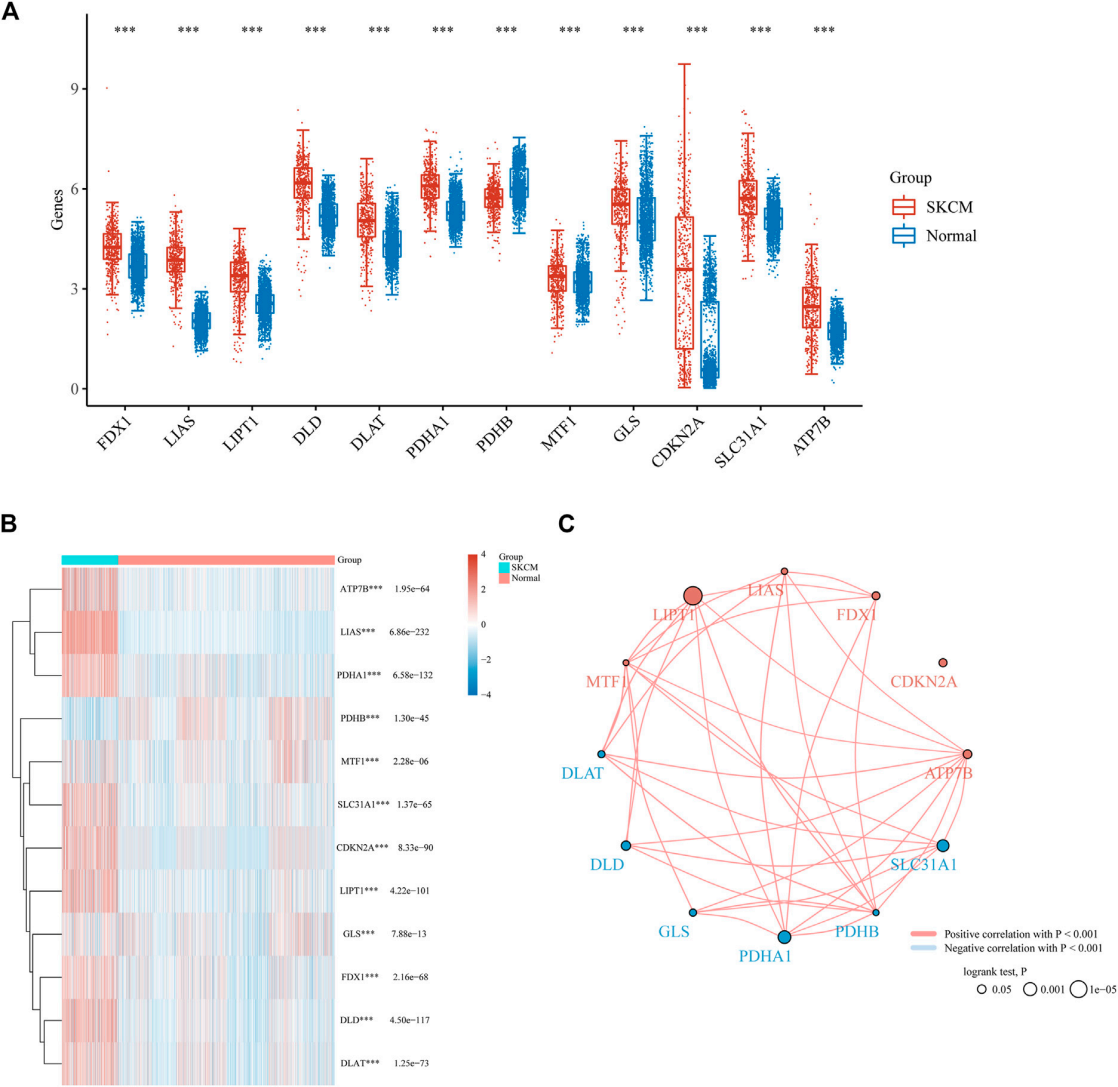
Cuproptosis-related genes expressed in SKCM patients and their correlation and prognostic value. Box plots **(A)** and heat map **(B)** of cuproptosis-related genes in SKCM compared to normal tissues. **(C)** Spearman correlation and prognostic values of cuproptosis-related genes in SKCM. Red represents positive correlation whereas blue represents negative correlation. The larger the circle the smaller log-rank *p*. ***p < 0.001.

As indicated previously, 12 genes (FDX1, LIAS, LIPT1, DLD, DLAT, PDHA1, PDHB, MTF1, GLS, CDKN2A, SLC31A1, and ATP7B) were demonstrated to be associated with cuproptosis (Tsvetkov et al., 2022). In order to confirm the involvement of these cuproptosis-related genes in melanoma, we compared the expression patterns of these 12 cuproptosis-related genes between melanoma and normal tissues downloaded from TCGA and GTEx databases. 470 cases of melanoma tissues and 1809 cases of normal tissues were enrolled in our analysis. Consequently, all these 12 genes were identified to be differentially expressed between melanoma and normal biopsies (Figures 1A,B). Among these differentially expressed genes, 11 genes (FDX1, LIAS, LIPT1, DLD, DLAT, PDHA1, MTF1, GLS, CDKN2A, SLC31A1, and ATP7B) were upregulated, while one gene (PDHB) were downregulated in melanoma (Figures 1A,B). The combination of correlation analysis and prognosis analysis indicated that the expression of most cuproptosis-related genes was positively correlated with each other, and three genes (LIPT1, PDHA1, and SLC31A1) had a prognostic value in melanoma (Figure 1C).

### Consensus Clustering Analysis of Cuproptosis-Related Genes Identified the Difference in Baseline Characteristics and Survival Between Two Melanoma Subgroups

**FIGURE 2:**
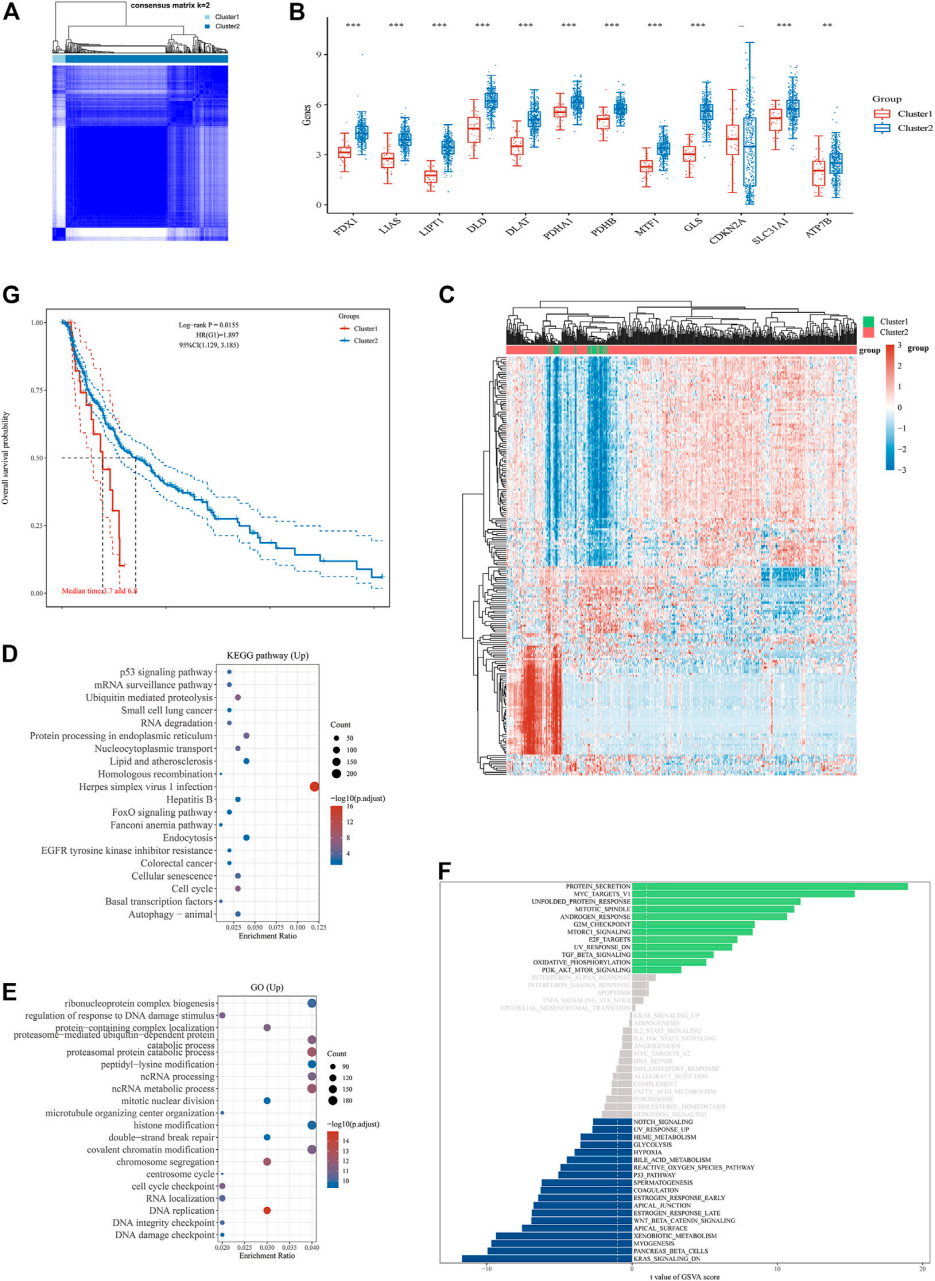
Differential expression pattern of cuproptosis-related genes and survival and functional enrichment analysis in two Skin Cutaneous Melanoma (SKCM) clusters. **(A)** Consensus clustering matrix for k = 2. **(B)** Box plots visualized the expression patterns of cuproptosis-related genes in two SKCM clusters. **(C)** The heatmap of the differential gene expression between two SKCM clusters. **(D–F)** The most significant KEGG pathways **(D)**, GO enrichment **(E)** and activation of multiple pathways by GSVA **(F)** in two SKCM clusters. **(G)** The Kaplan–Meier curves show the overall survival for two clusters of SKCM patients. **p < 0.01, and ***p < 0.001.

The consensus clustering analysis was conducted using the Consensus Cluster Plus package in R software. According to the expression of cuproptosis-related genes, the optimal clustering stability was chosen to be k = 2 in consensus clustering (Figure 2A, Supplementary Figure S2B). Consequently, the 470 melanoma patients downloaded from the TCGA database were divided into two subgroups, i.e., cluster 1 (high expression of cuproptosis-related gene group, *n* = 35) and cluster 2 (low expression of cuproptosis-related gene group, *n* = 435). The expressions of 11 cuproptosis-related genes (FDX1, LIAS, LIPT1, DLD, DLAT, PDHA1, PDHB, MTF1, GLS, SLC31A1, and ATP7B) were found to be higher in cluster two than cluster 1, while CDKN2A was not differentially expressed between cluster one and cluster 2 (Figure 2B). No cuproptosis-related genes were found to be lower expressed in cluster 2 as compared to cluster 1. In the bioinformatics analysis, 4,851 differentially expressed genes were found between cluster one and cluster 2. In contrast to cluster 1, 4,602 genes were upregulated, while 249 genes were downregulated in cluster 2 (Figure 2C). The enrichment analysis of the cluster two upregulated genes in the KEGG dataset found the enrichment in some pathways, including the p53 signaling pathway, cell cycle, cellular senescence, autophagy-animal, endocytosis, EGFR tyrosine kinase inhibitor resistance, ubiquitin-mediated proteolysis, protein processing in the endoplasmic reticulum, FoxO signaling pathway and so on (Figure 2D). Moreover, enrichment analysis of these genes in the GO database indicated the enrichment in some molecular processes, including regulation of response to DNA damage stimuli, DNA damage checkpoint, DNA integrity checkpoint, cell cycle checkpoint, chromosome segregation, histone modification, proteasomal protein catabolic processes, proteasome-mediated ubiquitin-dependent protein catabolic process, protein-containing complex localization and so on (Figure 2E). Additionally, the GSVA of these upregulated genes found the enrichment in diverse pathways in the Hallmark database, such as protein secretion, Myc targets, unfolded protein response, G2M checkpoint, PI3K/AKT/mTOR signaling, KRAS signaling, p53 pathway, and so on (Figure 2F). In addition, patients in cluster two showed much longer overall survival than patients in cluster 1 (Figure 2G). These findings indicated that this cuproptosis-related gene might inhibit the progression of melanoma by regulating immune-related and cell death-related molecular processes and pathways.

### Confirmation of LIPT1 Expression in Melanoma Patients and Bioinformatics Analysis of LIPT1-Related Gene Signatures

**FIGURE 3:**
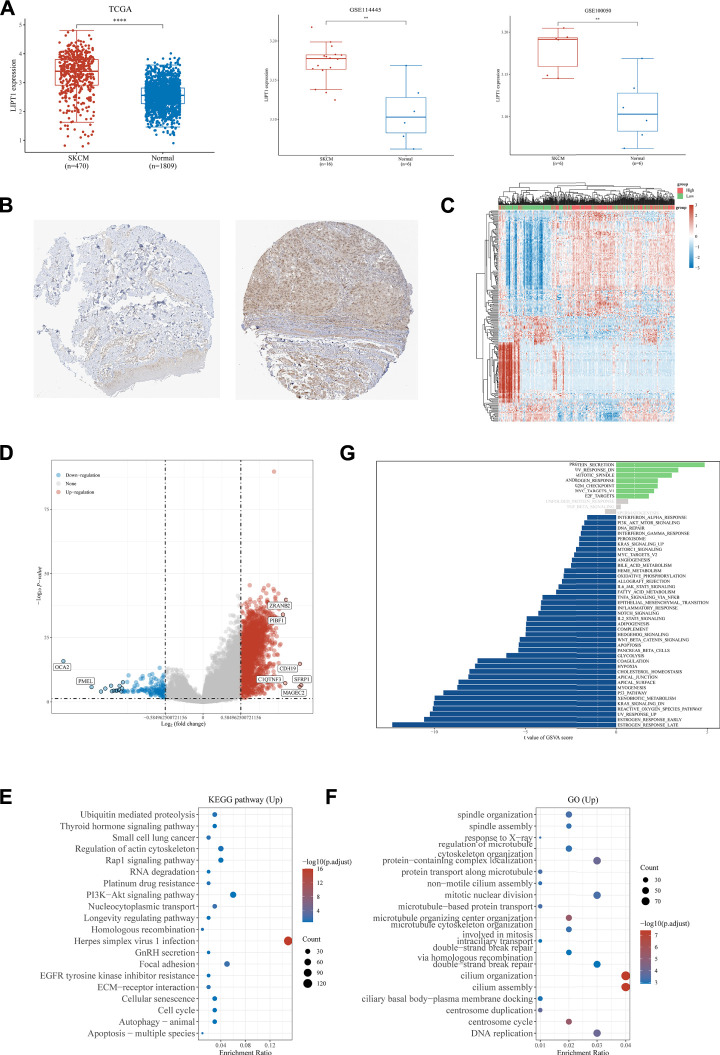
Analysis of the LIPT1 gene expression in SKCM. **(A)** Compared to normal tissues, LIPT1 was upregulated in SKCM tissues in the TCGA database, GSE114445, and GSE100050. **(B)** Immunohistochemistry analysis showed that LIPT1 was highly expressed in SKCM tissue (right) compared with normal skin tissue (left) in the human protein atlas (antibody HPA034802). The heatmap **(C)** and Volcano plot **(D)** of the differential gene expression between high and low expressed LIPT1 in SKCM. **(E,F)** The most significant KEGG pathways **(E)** and GO enrichment **(F)** between high and low expressed LIPT1 in SKCM. **(G)** Up and downregulation of LIPT1 was associated with activation of multiple pathways by GSVA. **p < 0.01 and ****p < 0.0001.

Lipoyltransferase 1, encoded by the LIPT1 gene, is involved in the metabolism of lipoic acid (Habarou et al., 2017; Tsvetkov et al., 2022). Mutations in LIPT1 were found to cause some inherited metabolic disorders, such as a Leigh disease with secondary deficiency for pyruvate and alpha-ketoglutarate dehydrogenase and a fatal disease related to a specific lipoylation defect of the 2-ketoacid dehydrogenase complexes (Soreze et al., 2013; Tort et al., 2014). In our present study, we found that LIPT1 was upregulated in melanoma patients as compared to normal tissues when analyzing the data downloaded from TCGA and GTEx databases (Figure 3A). We, therefore, chose LIPT1 to further explore its role in the development of melanoma. We downloaded and analyzed the expression of LIPT1 in sixteen melanoma and six normal tissues (GSE114445), and six melanoma and six normal tissues (GSE100050), respectively, to further confirm the differential expression of LIPT1 between melanoma and normal tissues. As shown in Figure 3A, the mRNA levels of LIPT1 were verified to be significantly higher in melanoma than in normal tissues in data downloaded from both GSE114445 and GSE100050 (Figure 3A). We next compared the protein levels of LIPT1 in melanoma and normal tissues downloaded from the Human Protein Atlas database and found that LIPT1 protein levels were dramatically upregulated in melanoma tissues as compared to normal tissues (Figure 3B). These findings suggested that LIPT1 might contribute to the development of melanoma.

To confirm the involvement of LIPT1 in melanoma, we divided the melanoma patients into two subgroups according to the expression of LIPT1, namely, the LIPT1-high group and the LIPT1-low group. In contrast to the LIPT1-low group, 2,174 upregulated genes (such as ZRANB2, PIBF1, CDH19, C1QTNF3, SFRP1, and MAGEC2) and 145 downregulated genes were identified in LIPT1-high group (such as OCA2 and PMEL) (Figures 3C,D). The enrichment analysis of the upregulated genes in the KEGG dataset revealed some cell death and immune response-related pathways, such as PI3K-AKT signaling pathway, longevity regulating pathway, cellular senescence, cell cycle, autophagy-animal, apoptosis-multiple species, EGFR tyrosine kinase inhibitor resistance, ubiquitin-mediated proteolysis, ECM-receptor interaction and so on (Figure 3E). The further enrichment analysis of these upregulated genes in the GO dataset indicated some molecular processes, such as double-strand break repair *via* homologous recombination, double-strand break repair, DNA replication, centrosome cycle, and so on (Figure 3F). The following GSVA of the upregulated genes indicated the enrichment in various pathways, including reactive oxygen species pathway, hypoxia, oxidative phosphorylation, peroxisome, fatty acid metabolism, glycolysis, PI3K/AKT/mTOR signaling, KRAS signaling, p53 pathway, apoptosis, G2M checkpoint, angiogenesis, interferon-alpha response, interferon-gamma response, IL6/JAK/STAT3 signaling, TNFαsignaling *via* NFKB, IL2/STAT5 signaling, complement, inflammatory response and so on, which were involved in metabolism, cell death and immune responses (Figure 3G).

### Evaluation of Prognostic Value of LIPT1 in Melanoma Patients

**FIGURE 4:**
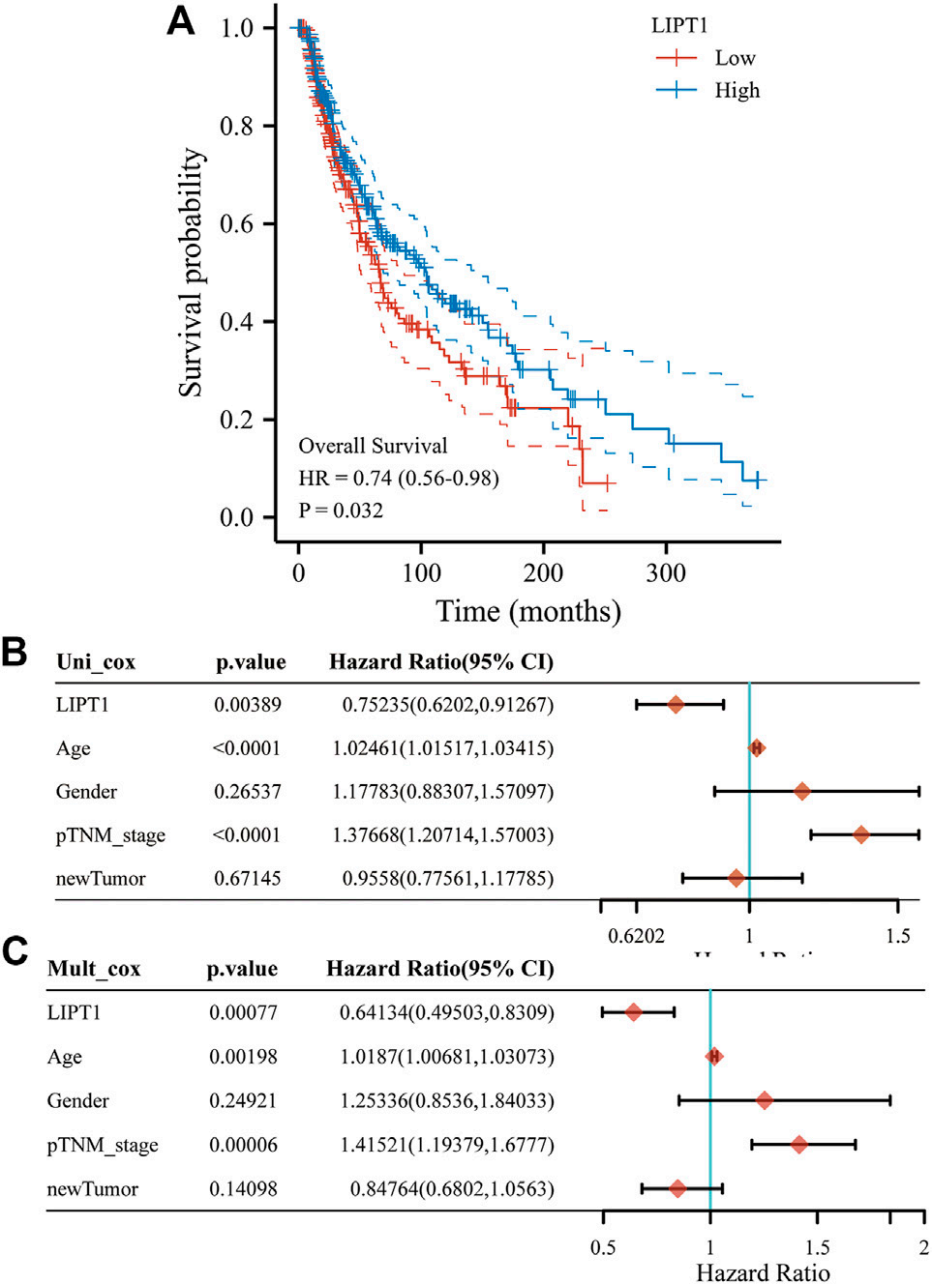
Upregulated LIPT1 expression is connection with good outcomes of Skin Cutaneous Melanoma (SKCM). **(A)** The Kaplan–Meier analysis of SKCM patients with high and low LIPT1 expression level in the TCGA cohort. **(B,C)** Univariate **(B)** and multivariate Cox analysis of expression of LIPT1 and other factors in SKCM patients.

The prognostic value of upregulated LIPT1 in melanoma was further evaluated. The melanoma patients who expressed higher LIPT1 showed longer overall survival than those who expressed lower LIPT1 (Figure 4A). Moreover, we checked the relationship between LIPT1 expression and overall survival in melanoma using the Cox analysis and the results were shown in the forest plots. As shown in Figure 4B, the univariate analysis indicated that LIPT1 expression (HR = 0.75235, *p* = 0.00389), age (HR = 1.02461, *p* < 0.0001) and TNM stage (HR = 1.37668, *p* < 0.0001) were correlated with overall survival of melanoma (Figure 4B). The multivariate analysis indicated that LIPT1 expression was an independent factor for predicting the progression of melanoma patients (HR = 0.64134, *p* = 0.00077, Figure 4C).

### Correlation Analysis of LIPT1 Expression With PD-L1 Levels and Immune Cell Infiltration

With the approval of ICIs for the treatment of melanoma, it became one of the most important therapies for melanoma patients since they could improve the overall survival and objective response rates in melanoma patients. PD-L1 is an essential marker for the prediction of the response to ICIs, and we confirmed that the expression of PD-L1 was higher in melanoma than in normal tissues (Supplementary Figure S2A). To investigate the relationship between the expression of PD-L1 and cuproptosis-related genes, we explored the expression of PD-L1 in both cluster one(lower expression of copper-reduced cell death-related genes) and cluster 2 (higher expression of cuproptosis-related genes) that downloaded from TCGA database and found higher expression of PD-L1 in the sub-group that expressed higher cuproptosis-related genes (Supplementary Figure S2B). Moreover, we detected that PD-L1 expression was positively correlated with some cuproptosis-related genes (CDKN2A, FDX1, LIPT1, and MTF1), but negatively correlated with other cuproptosis-related genes (ATP7B, DLST, and PDHA1) in an analysis of 470 melanoma patients (Supplementary Figure S2C). Moreover, our GSVA found the enrichment of LIPT1-higher group upregulated genes in many immune-related pathways, including IFNα signaling, IFNγ signaling, TNFα signaling, and JAK/STAT pathways that were found to be closely related to the response to immunotherapy in tumors. We, therefore, examined the role of LIPT1 in immunotherapy in melanoma patients.

**FIGURE 5:**
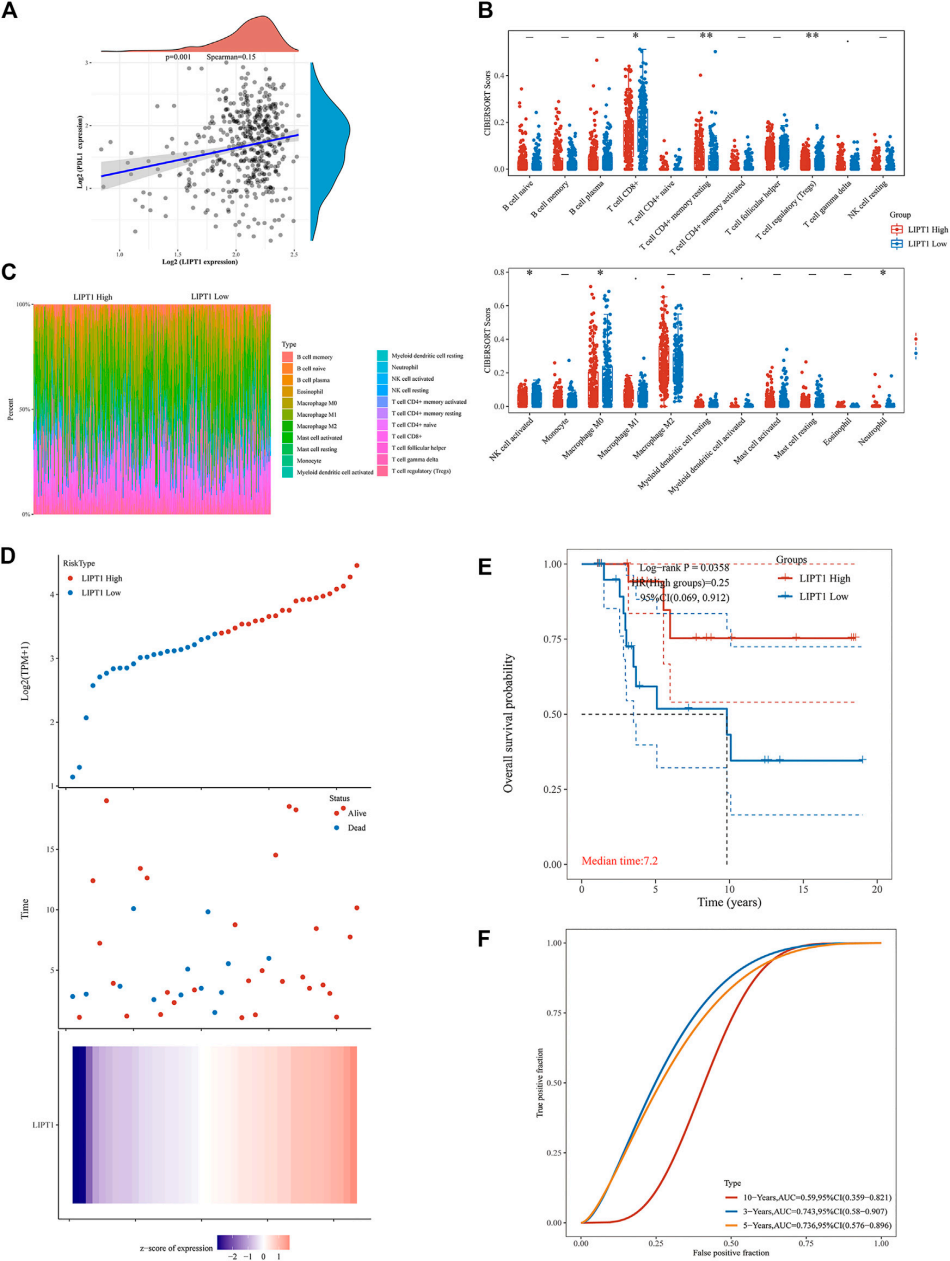
Relationship between LIPT1 expression, immunotherapy and infiltrating immune cells in Skin Cutaneous Melanoma (SKCM) by correlation analysis. **(A)** The correlation analysis of PD-L1 expression and LIPT1 expression in SKCM. **(B)** The infiltrating levels of immune cells in high and low LIPT1 expression groups in SKCM patients. **(C)** The percentage abundance of tumor-infiltrating immune cells showed the immune infiltration analysis between high and low LIPT1 expression groups in SKCM patients. **(D)** The gene expression, survival time, and survival status of the TCGA database. **(E)** Kaplan-Meier survival analysis of the gene signature from TCGA cohort. **(F)** The ROC curve of LIPT1.

We confirmed the relationship between LIPT1 and PD-L1 expression in 470 melanoma patients using Spearman correlation analysis and validated a positive correlation between LIPT1 and PD-L1 expression (*p* = 0.001, Spearman = 0.15, Figure 5A). Subsequently, we evaluated the correlation between LIPT1 expression and immune cell infiltration in these melanoma patients. The patients were classified into two subgroups (LIPT1-high and LIPT-low) according to LIPT1 expression. As shown in Figure 5B, differential infiltration of some immune cells was found between LIPT1-high and LIPT1-low groups. As compared to the LIPT1-low group, the infiltration of CD4^+^ memory T cells resting was higher, while the infiltration of CD8^+^ T cells, regulatory T cells (Tregs), activated NK cells, M0 macrophages, neutrophils were lower in LIPT1-high group (Figure 5B). The infiltration of these immune cells in each tumor sample in both subgroups was visualized in the heat map of tumor-infiltrating immune cells (Figure 5C).

### Evaluation of Prognostic Value of LIPT1 in Melanoma Patients Receiving Immunotherapy

In this part, we evaluated the prognostic value of upregulated LIPT1 in melanoma patients receiving immunotherapy. We divided the melanoma patients receiving immunotherapy downloaded from TCGA into two subgroups (LIPT1-high and LIPT1-low) according to the expression of LIPT1, and checked the survival status of these patients. As shown in Figure 5D, more patients were found to be alive in the LIPT1-high group as compared to the LIPT1-low group (Figure 5D). Additionally, the LIPT1-high group showed longer overall survival than the LIPT1-low group (Figure 5E). Subsequently, the efficiency of LIPT1 for predicting the prognosis of melanoma patients receiving immunotherapy was evaluated through receiver operating characteristic (ROC) curve analysis. The area under the curve (AUC) values for predicting 3-, 5, and 10-year survival were 0.743, 0.736, and 0.59, respectively (Figure 5F). The findings above indicated that high expression of LIPT1 might be an indicator of the favorable prognosis of melanoma after immunotherapy.

### Comprehensive Analysis of LIPT1 in Pan-Cancer

**FIGURE 6:**
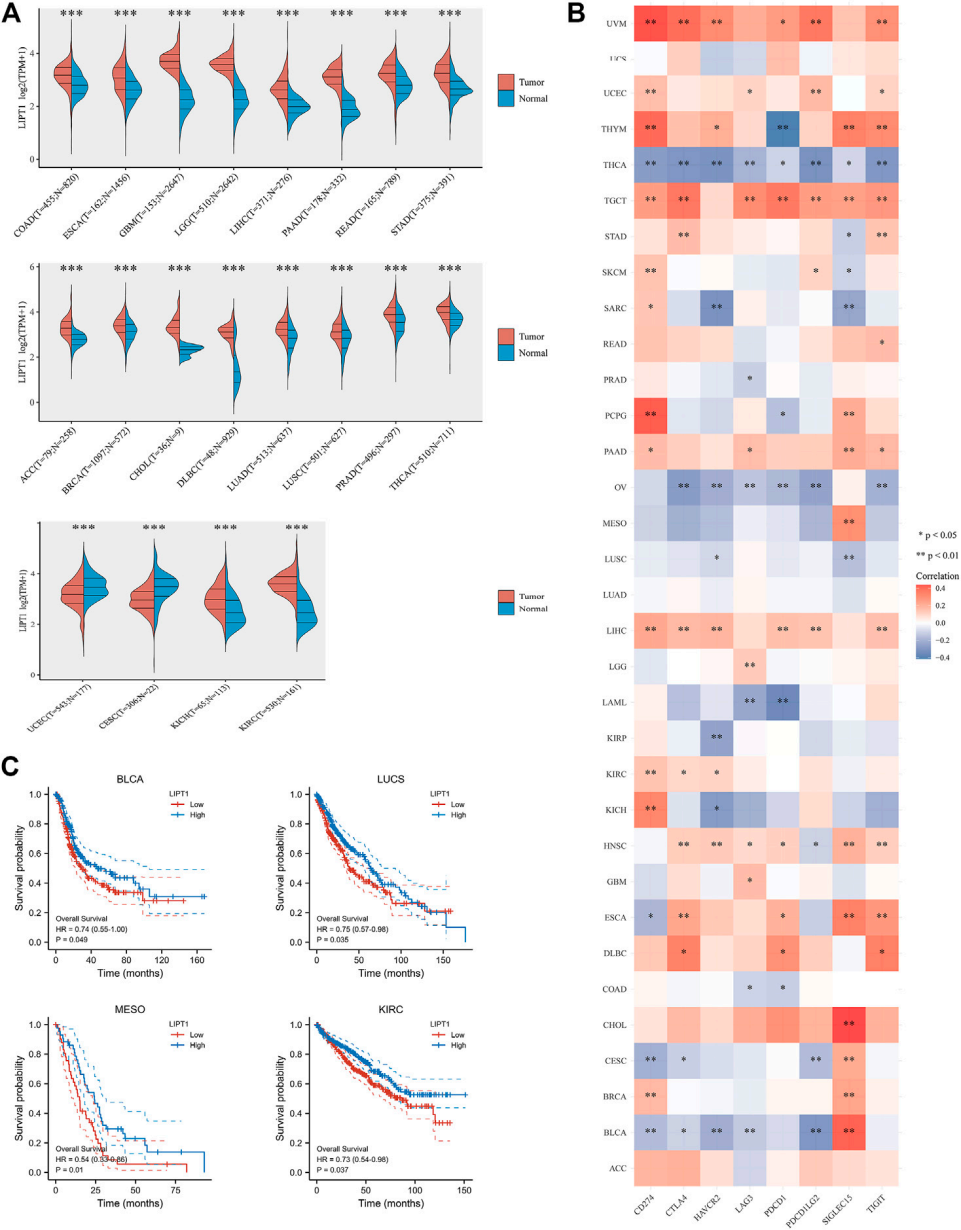
Comprehensive analysis of LIPT1 in pan-cancers. **(A)** LIPT1 was differentially expressed in most cancer types. **(C)** Highly expressed LIPT1 showed good overall survival in multiple cancers. **(B)** The relationship between expression of LIPT1 and eight common immune checkpoints in pan-cancers. *p < 0.05, **p < 0.01, and ***p < 0.001.

Since immune checkpoint blockade therapy has been approved for the treatment of some other tumors, besides melanoma, and achieved great success. In this part, we determined to examine whether LIPT1 expression could be a potential predictor for the prognosis of other types of tumors. First, we compared the expression of LIPT1 in different kinds of tumors and corresponding normal tissues downloaded from TCGA and GTEx databases. As shown in Figure 6A, the mRNA levels of LIPT1 were found to be upregulated in 18 different tumor tissues (COAD, ESCA, GBM, LGG, LIHC, PAAD, READ, STAD, ACC, BRCA, CHOL, DLBC, LUAD, LUSC, PRAD, THCA, KICH, and KIRC), while downregulated in two different tumors (CESC, UCEC), as compared to normal tissues (Figure 6A). The prognostic value of LIPT1 in these cancers was subsequently evaluated. We divided the tumor patients into two subgroups according to the expression of LIPT1, namely, the LIPT1-high and LIPT1-low groups. As shown in Figure 6C, LIPT1-high groups showed longer overall survival than LIPT1-low groups in BLCA, LUCS, MESO, and KIRC, respectively (Figure 6C). We further explored the correlation between the expression of LIPT1 and eight immune checkpoints (PD-L1, CTLA4, HAVCR2, LAG3, PDCD1, PDCD1LG2, SIGLEC15, and TIGIT), and found that LIPT1 levels were correlated with the expressions of these immune checkpoints, especially PD-L1, in most cancer types (Figure 6B). Therefore, LIPT1 had the potential in regulating and predicting the response to immune checkpoint blockade therapies in some kinds of tumors.

## Discussion

Melanoma is the most lethal skin cancer with increasing incidence worldwide over the past several decades (Muzumdar et al., 2021; Welch et al., 2021; Huang and Zappasodi, 2022). It has multiple risk factors involved in the interaction between genetic susceptibility and environmental exposure (Rastrelli et al., 2014). Its treatment remains challengeable since it showed poor response to the traditional chemotherapy (Chapman et al., 2011). Although the development of target therapies and immune checkpoint blockade therapies has benefited malignant melanoma patients a lot, the primary and acquired resistance remains a big problem (Sosman et al., 2012; Postow et al., 2015). Therefore, a comprehensive understanding of the genetic backgrounds and tumor microenvironment is ultimate for the prevention, treatment, and prognosis evaluation of melanoma.

The large-scale public database, such as the Cancer Genome Atlas (TCGA) and Gene Expression Omnibus (GEO), presented the transcriptome profiles of numerous cancers. The access and analysis of this large-scale database allowed us to have an overall view of the genetic landscape, identify new biomarkers, screen therapeutic strategies and predict prognosis of these cancers (Yan et al., 2018; Zhou et al., 2019; Wang et al., 2021).

RCD plays essential role in numerous pathological and physiologic processes, including tumorigenesis (Tang et al., 2019). As the best-studied RCD form, apoptosis serves as the target of nearly all tumor treatment strategies (Tang et al., 2019; Qi et al., 2022). However, apoptosis resistance may cause the failure of these treatments (Gong et al., 2019). Therefore, it is necessary to find out more about RCD forms and have a deeper understanding of the roles of different RCD forms in tumorigenesis. Ferroptosis is a novel iron-dependent lipid peroxidation induced RCD that is different from other RCD forms, such as apoptosis, necroptosis, and autophagy (Lei et al., 2020). Disruption of ferroptosis has been linked with tumorigenesis (Stockwell et al., 2017; Ding et al., 2021). Evaluation of the prognostic value of ferroptosis regulated genes has been widely conducted in diverse tumors, such as melanoma, clear cell renal cell carcinoma, breast cancer, lung adenocarcinoma, hepatocellular carcinoma, and so on (Liang et al., 2020; Jin et al., 2021; Li et al., 2021; Wang et al., 2021; Ping et al., 2022). The correlation between ferroptosis regulators and immune signatures has also been explored in different tumors, including melanoma (Fan et al., 2021; Wang et al., 2021; Liu et al., 2022). Dividing tumor patients into subgroups based on their molecular signatures, such as ferroptosis-related gene signatures, enable us to predict their diverse phenotypes, treatment response, and prognosis (Wang et al., 2021).

Cuproptosis is a newly revealed RCD form that is distinct from other RCD forms, such as apoptosis, ferroptosis and necroptosis. It is dependent on mitochondrial respiration (Tsvetkov et al., 2022). As a kind of RCD, the role of cuproptosis in tumorigenesis remains unknown. In our present study, we first evaluated the prognostic value of cuproptosis-related gene signatures in melanoma. We found that 11 out of 12 cuproptosis-related genes were upregulated, while 1 out of 12 genes was downregulated in melanoma as compared to normal tissues. We next divided the melanoma patients into two subgroups according to the expression of cuproptosis-related genes and found the melanoma patients who expressed higher cuproptosis-related genes showed longer overall survival than those with lower cuproptosis-related gene expression. Therefore, cuproptosis-related gene signatures are potential indicators for the prognosis of melanoma.

As an important trace element, the role of copper in tumorigenesis and tumor treatment has been studied a lot. However, it remains contradictory. For example, serum copper levels were found to be elevated and able to reflect the degree of tumor activity in melanoma patients (Fisher et al., 1981). Copper has further been demonstrated to contribute to tumorigenesis through binding to MEK1 and forming a copper-MEK1 interaction. Inhibiting the expression of CTR1 or mutations in MEK1 that disrupt the binding, could inhibit BRAF signaling and tumorigenesis (Brady et al., 2014). Therefore, copper serves as an important target for cancer therapy. Disruption of copper import genetically or inhibition of copper concentration pharmacologically with a copper chelator, has been demonstrated to inhibit BRAF-driven melanomagenesis and overcome BRAF or MEK1/2 inhibitor resistance through inhibiting MEK1/2 activity (Brady et al., 2014; Brady et al., 2017). However, metallic copper nanoparticles were identified to induce a series of pathological processes, including cell membrane rigidity abating, DNA degradation, chromosome condensation, cell cycle arrest in the G2/M phase, mitochondrial membrane depolarization, oxidative stress induction, and apoptosis, thus leading to cell death in melanoma cell lines (Chakraborty and Basu, 2017; Mukhopadhyay et al., 2018).

As a novel RCD form, cuproptosis is dependent on the binding of copper to components of the TCA cycle directly instead of the electron transcript chain, subsequently reducing the spare capacity of mitochondrial respiration (Tsvetkov et al., 2022). Knockout of cuproptosis genes, including FDX1, LIPT1, LIAS, DLD, DLAT, PDHA1, and PDHB, could rescue cuproptosis. The TCA cycle plays a pivotal role in oxidative metabolism. It provides carbon for biosynthesis and reducing agents for ATP generation. In hypoxia, melanoma cells maintain proliferation by reversely running the TCA cycle (Filipp et al., 2012). We hypothesized that disruption of the TCA cycle and subsequent mitochondrial respiration by excessive copper may inhibit the proliferation of melanoma cells, thus inhibiting tumor growth. Therefore, cuproptosis may be a potential target for melanoma treatment, and cuproptosis-related gene signatures may be a new predictor for the genotype, therapeutic response, and prognosis in melanoma, which provided new clues for investigating the role of copper in melanomagensis. Sincerely, more research are needed to further confirm this hypothesis.

The further bioinformatic analysis of the upregulated genes in the subgroup expressed higher cuproptosis-related genes found the enrichment of some cell death and immune response-related pathways. These pathways, such as the p53 signaling pathway, cell cycle, PI3K/AKT/mTOR signaling, and KRAS signaling have been widely confirmed to be involved in melanoma. Therefore, these upregulated cuproptosis-related genes might inhibit the progression of melanoma by mediating these classical pathways associated with cell death and immune responses.

Lipoic acid is a pivotal cofactor for the 2-ketoacid dehydrogenases (pyruvate dehydrogenase complex, 2-oxoadipate dehydrogenase, α-ketoglutarate dehydrogenase, branched-chain ketoacid dehydrogenase) and the glycine cleavage system in mitochondria which are related to TCA cycle and mitochondrial energy metabolism and amino acid catabolism (Hiltunen et al., 2010; Mayr et al., 2014; Stowe et al., 2018; Solmonson et al., 2022). Lipoyltransferase 1, encoded by LIPT1, transferred lipoate to the E2 subunits of the 2-ketoacid dehydrogenases (Hiltunen et al., 2010; Stowe et al., 2018). Thus, deficiency in the LIPT1 homolog could lead to decreased lipoylation of the E2 subunits (Schonauer et al., 2009; Stowe et al., 2018). LIPT1 deficiency was also demonstrated to suppress TCA cycle metabolism (Solmonson et al., 2022). LIPT1 has further been revealed to support lipogenesis and balance oxidative and reductive glutamine metabolism in the functional assessment (Ni et al., 2019). LIPT1 deficiency was reported in embryonic demise, early death or Leigh-like encephalopathy, such as early infantile epileptic encephalopathy, Leigh disease, secondary pyruvate dehydrogenase complex deficiency, fatal lactic acidosis (Soreze et al., 2013; Habarou et al., 2017; Stowe et al., 2018; Solmonson et al., 2022). However, the role of LIPT1 in the tumorigenesis and progression of tumors is far to be known. Yuanbin et al. found that LIPT1 was among the genes that were correlated with a favorable prognosis of urothelial cancer patients through analysis of the Pathology Atlas (Chen et al., 2021). Overexpression of LIPT1 in bladder cancer cell lines could, to some extent, inhibit cell migration, while having no effect on cell viability (Chen et al., 2021). However, the mechanism underlying the regulation of LIPT1 on cell migration remains unknown.

In our present study, we found that LIPT1 was upregulated in melanoma by analyzing the data downloaded from the TCGA and GEO databases, respectively. We further found that LIPT1 could be an indicator of the favorable prognosis of melanoma. Therefore, we hypothesized that upregulated LIPT1 might inhibit tumorigenesis and progression by disrupting TCA in mitochondria and subsequently inducing cuproptosis. Since the further GSVA of LIPT1 upregulated genes revealed the enrichment in some metabolism and cell death-related pathways, upregulated LIPT1 might inhibit tumor cell proliferation by inducing other forms of RCD.

Moreover, we found that LIPT1 expression was positively correlated with PDL1 expression and negatively associated with the infiltration of Tregs. LIPT1 could be a positive indicator for the prognosis of melanoma patients receiving immunotherapy. Since the GSVA of LIPT1 upregulated genes revealed the enrichment in some immune response-related pathways, LIPT1 might regulate these pathways, such as IFNγ and IFNα to enhance the response to immunotherapy.

Regulatory T (Treg) cells, characterized by the expression of Foxp3, are required for the maintaining of self-tolerance through inhibiting immune responses to self and nonself antigens in an antigen non-specific way (Sakaguchi et al., 1995; Fontenot et al., 2005; Overacre-Delgoffe et al., 2017). The lack of Tregs leads to evident autoimmune disease (Sakaguchi et al., 1995; Overacre-Delgoffe et al., 2017). However, tumor-infiltrating Tregs are found to be related to poor clinical outcomes. They promoted cancer progression because of the ability to inhibit antitumor immunity (Facciabene et al., 2012). Therefore, they are regarded as a major barrier to the successful application of immunotherapy (Liu et al., 2016; Arce Vargas et al., 2017). Depletion of tumor-infiltrating Tregs was found to show synergistic effects with ICI to eradicate tumors (Arce Vargas et al., 2017; Van Damme et al., 2021). In our present study, we found that LIPT1 expression was negatively correlated with the infiltration of Tregs. Therefore, upregulated LIPT1 might improve the response to immunotherapy by inhibiting Treg infiltration in the tumor microenvironment.

IFNγ, an important cytokine mainly secreted by activated T cells, plays an important antitumor effect in multiple ways. For example, it promotes the cytotoxic effect of effective CD8^+^ T cells and inhibits the regulatory activity of Tregs (Kursunel and Esendagli, 2016; Giunta et al., 2020). The IFNγ signaling signatures has also been found to be highly correlated with the response to ICI in diverse tumors, including melanoma (Ayers et al., 2017; Grasso et al., 2020). Activation of the IFNγ signaling could improve the response to ICI, while inhibition of the IFNγ signaling could induce resistance to ICI in these tumors (Kim et al., 2021; Nguyen et al., 2021). In our present study, we found the enrichment of LIPT1 upregulated genes in some immune response-related pathways, including IFNγ signaling pathway. Therefore, LIPT1 might improve the therapeutic benefits of immunotherapy thorough activating IFNγ signaling pathway.

In conclusion, we found that 11 out of 12 genes were upregulated in melanoma and 3 out of 12 genes have predictive value for the prognosis. Furthermore, the upregulated cuproptosis-related gene, LIPT1, was positively correlated with PD-L1 expression and negatively correlated with Treg infiltration. It could be a predictor of the prognosis in melanoma patients receiving immunotherapy.

## Data Availability

The datasets presented in this study can be found in online repositories. The names of the repository/repositories and accession number(s) can be found in the article/Supplementary Material.
