# Supplementary material for: Comprehensive Analysis of Cuproptosis-Related Genes in Immune Infiltration and Prognosis in Melanoma
Source: Front Pharmacol. 2022 Jun 28;13:930041. doi: 10.3389/fphar.2022.930041 (PMC9273972; doi:10.3389/fphar.2022.930041)
Supplement: Supplementary file 1 [file DataSheet1.docx]

Supplementary Material


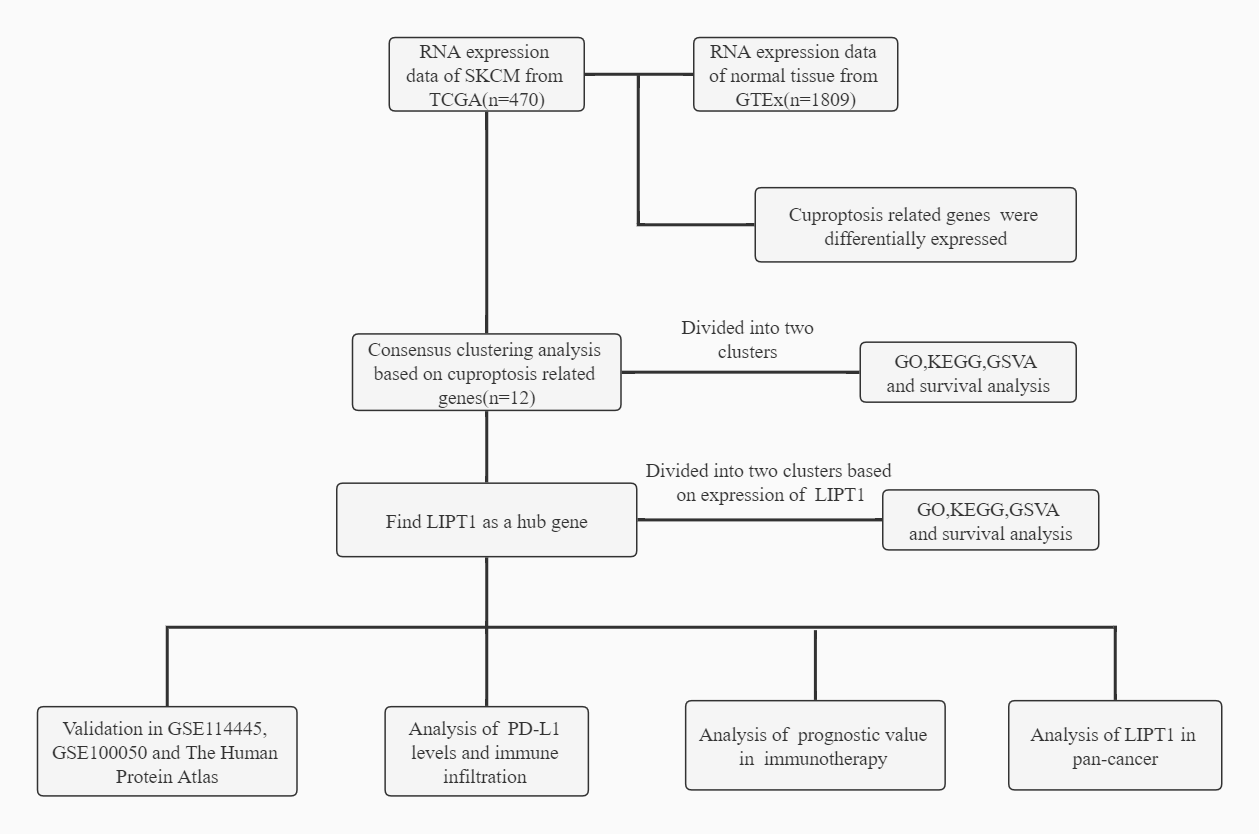


**Supplementary Figure 1.** Schematic depicting our study design.


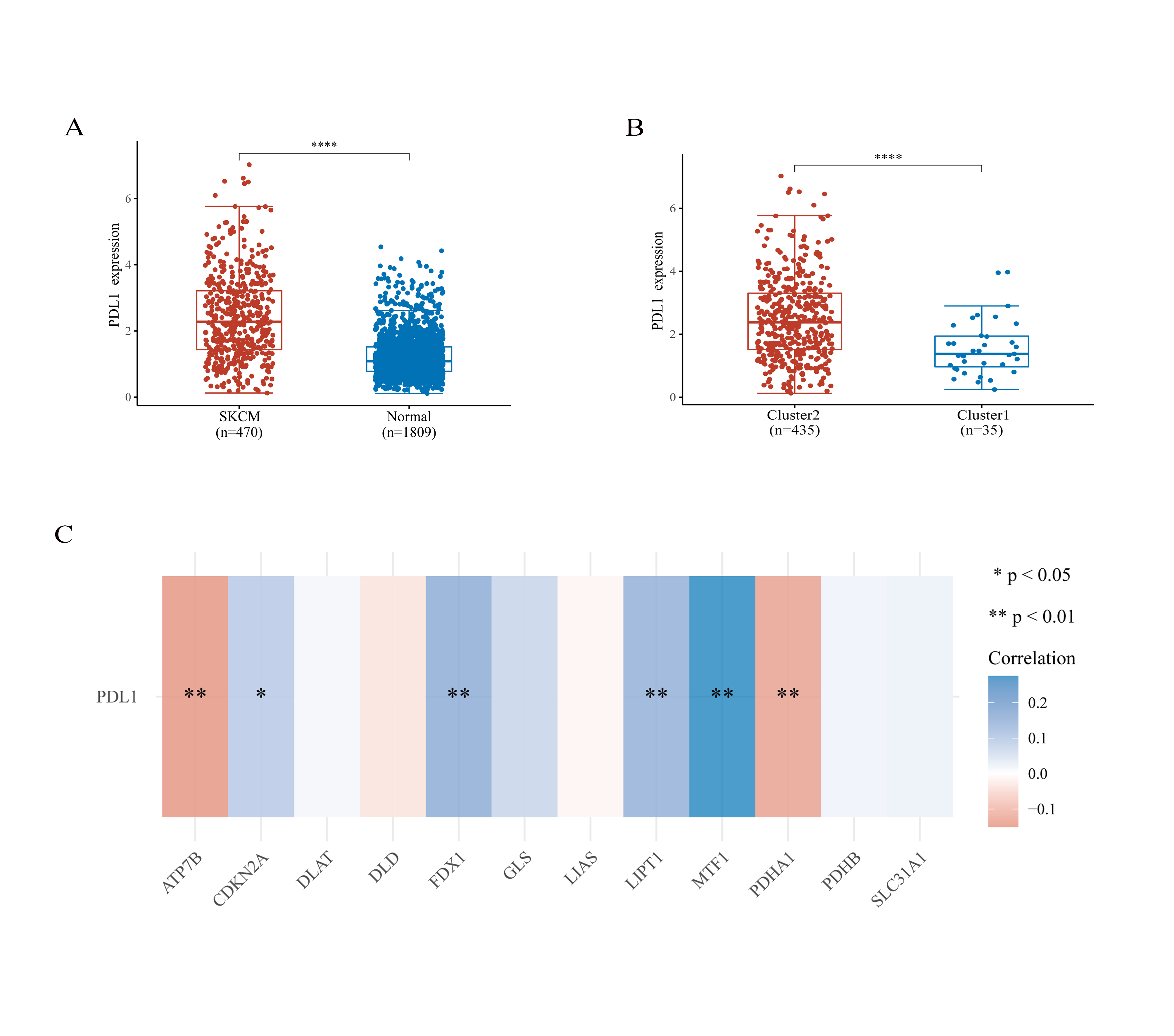


**Supplementary Figure 2.** Correlation between PD-L1 and cuproptosis related genes in Skin Cutaneous Melanoma (SKCM). (A,B) The expression level of PD-L1 in SKCM/normal group (A) and cluster 1/2 (B) in SKCM. (C) The correlation of PD-L1 with cuproptosis related genes in the SKCM.


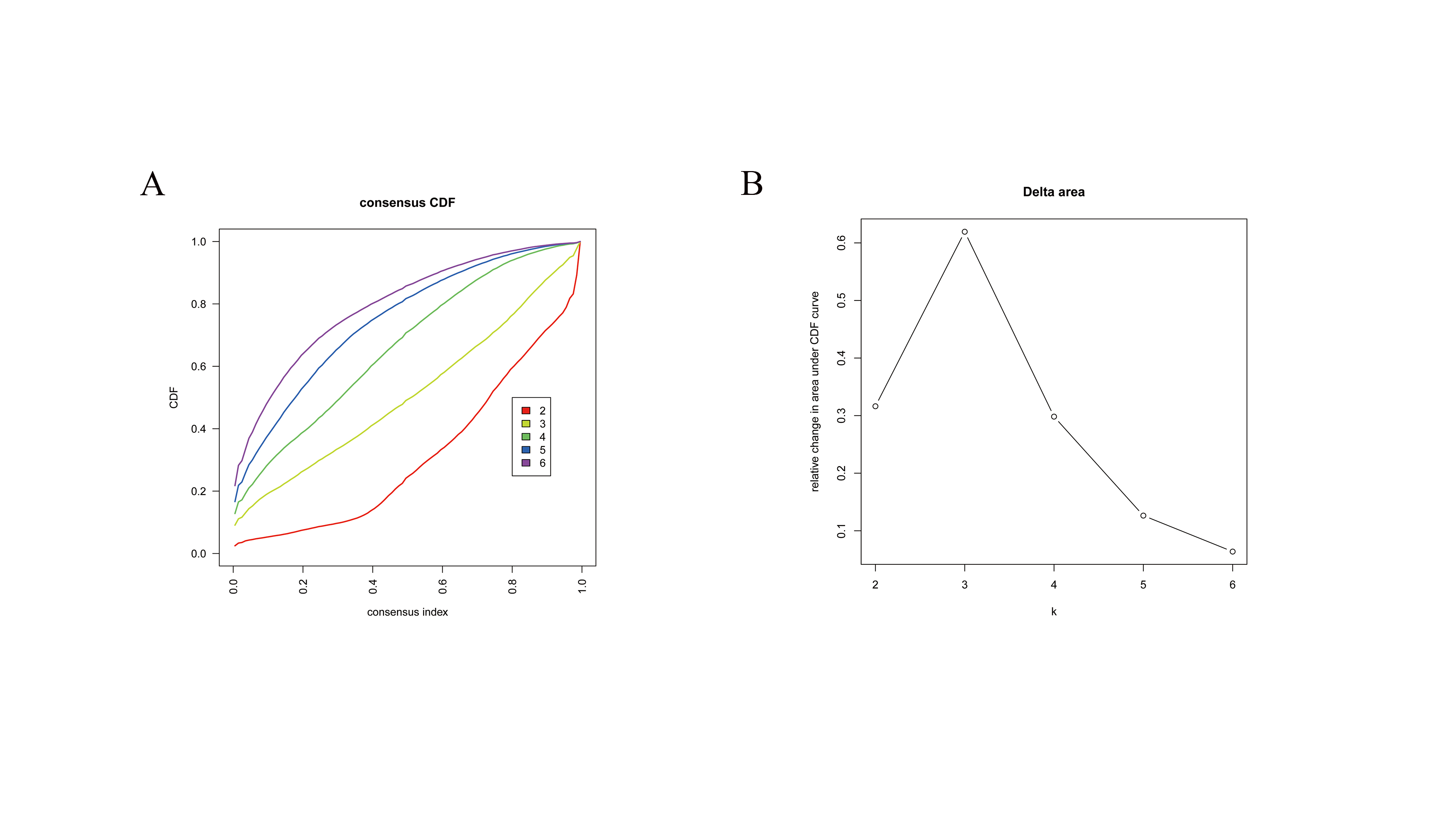


**Supplementary Figure 3**. Consensus clustering applied on cuproptosis related genes. (A,B) Cumulative distribution function curves for k=2–6.
